# There and back again: consequences of biofilm specialization under selection for dispersal

**DOI:** 10.3389/fgene.2015.00018

**Published:** 2015-02-11

**Authors:** Devon O’Rourke, Cody E. FitzGerald, Charles C. Traverse, Vaughn S. Cooper

**Affiliations:** ^1^Molecular, Cellular, and Biomedical Sciences, University of New HampshireDurham, NH, USA; ^2^Department of Microbiology, University of Texas at AustinAustin, TX, USA

**Keywords:** biofilm, *Burkholderia cepacia*, dispersal, adaptation, evolution, exopolymeric substances, contingency

## Abstract

Experimental evolution paired with modern sequencing can be a powerful approach to identify the mechanisms by which bacteria adapt to discrete environmental conditions found in nature or during infections. We used this approach to identify mechanisms enabling biofilm specialists of the opportunistic respiratory pathogen *Burkholderia cenocepacia* to regain planktonic fitness. Seven mutants producing wrinkly (W) small-colony variants by mutations in the wrinkly-spreader operon (*wsp*) cluster, but with varying duration of biofilm adaptation, served as ancestors of this experiment. Following planktonic growth, each W ancestor produced smooth (S) mutants with distinct fitness effects across planktonic, biofilm, and dispersal-phase environments. The causes of the S phenotype traced to mutations in three gene clusters: *wsp*, *Bcen2424_1436*, an uncharacterized two-component transcriptional regulator which appears to be critical for *wsp* signaling, and a cohort of genes involved in polysaccharide synthesis. The genetic pathway from W to S also associated with evolutionary history in the biofilm environment. W mutants isolated from long-term biofilm selection usually produced S types via secondary *wsp* mutations, whereas S types evolved from less adapted W ancestors by a wider scope of mutations. These different genetic pathways to suppress the W phenotype suggest that prolonged biofilm adaptation limits routes to subsequent planktonic adaptation, despite common initial mechanisms of biofilm adaptation. More generally, experimental evolution can be used as a nuanced screen for gain-of-function mutations in multiple conditions that illustrate tensions that bacteria may face in changing environments or hosts.

## INTRODUCTION

Two bedrock techniques of genetic analysis are the mutant screen for a particular novel phenotype and the suppressor screen for mutants reverting in phenotype. Commonly these methods have involved transposon or chemical mutagenesis, which may produce severe and even unrealistic mutations that hamper or eliminate the possibility of reversion. Today, combining experimental evolution with modern sequencing can accomplish these same goals with potentially greater realism and sample a broader spectrum of genetic variation (Elena and Lenski, 2003; Kawecki et al., 2012; Barrick and Lenski, 2013). Moreover, the kinds of mutations captured by experimental evolution can produce insights into the function of the altered transcript. For instance, gain-of-function mutations in hypothetical open reading frames can chart a short path to identifying the function of an undescribed gene, and the location of mutations within a known gene can demonstrate the relative importance of different domains for a particular function. Put another way, the evolution of several non-synonymous mutations in an unstudied genome region that are associated with adaptation can illustrate more about the relevant environmental pressures on bacterial physiology than the effects of a loss-of-function (LOF) gene knockout. One added benefit of experimental evolution is that the trajectories of contending mutations can explain the relative strength of selection for that phenotype (Cooper, 2007; Lang et al., 2013; Traverse et al., 2013), although this study focuses on clones representing certain contending mutants.

One environmental transition that likely generates significant selection for many bacteria is the switch from surface attachment to dispersal in a planktonic environment. Life on surfaces, or in biofilms, may dominate the lifestyle of some species in certain environments, but appear less critical in other environments that enable more rapid growth in mass-action conditions. The means by which bacteria adapt to changing conditions that favor either biofilm formation or dispersal underlie a variety of important processes, such as the development of antimicrobial resistance (Jeannot et al., 2008; Guo and Rowe-Magnus, 2010; Sawasdidoln et al., 2010; Lee et al., 2013), the construction of durable microbial communities, and colonization of new environments or hosts (Danhorn and Fuqua, 2007; Lee et al., 2013). Although many mechanisms involved in biofilm formation have been defined for a range of species (Abee et al., 2011; Fazli et al., 2014), research describing the genetic and physiological paths regulating dispersal is less comprehensive, as reviewed by (Kaplan, 2010; McDougald et al., 2012).

Recently, we explored the genetic causes of Wrinkly (W) small colony variants (SCVs) in the biofilm-forming species, *Burkholderia cenocepacia*, using experimental evolution as a screen for these adaptive phenotypes in a laboratory model of biofilm selection (Poltak and Cooper, 2011; Traverse et al., 2013). *Burkholderia* SCVs are associated with worse patient prognoses during pulmonary infections of persons with cystic fibrosis (CF) (Haussler et al., 2003; Silva et al., 2011), and correlate with higher biofilm productivity in our laboratory model (Poltak and Cooper, 2011). This model of selection involves a daily cycle of attachment to a polystyrene bead, biofilm formation, dispersal, and reattachment (Poltak and Cooper, 2011), and W colonies are among the first variants to evolve in each replicate population. We quantified relative fitness and biofilm phenotypes of many W mutants and found them to be uniformly highly adaptive, and sequenced their genomes to determine their genetic bases (Cooper et al., 2014). The sequences revealed exceptional parallelism in mutations affecting the operon orthologous to the well-characterized *Pseudomonas wsp* (wrinkly-spreader) operon: 29 of 35 causative, non-synonymous mutations affected the sensor protein, WspA, or the terminal sensor histidine kinase, WspE (Bantinaki et al., 2007; Cooper et al., 2014). Identical missense mutations occurred in multiple replicates and highlighted residues important for system function. This study implied that the missense mutations led to constitutive activation of signaling in contrast to the LOF mutations that de-repress the system observed in *Pseudomonas* (McDonald et al., 2009). This study also showed that different W mutants inhabit a complementary niche by attaching early to the plastic surface and facilitating attachment of other cells (Traverse et al., 2013; Cooper et al., 2014). Nevertheless, the proximate, structural cause of the W phenotype and its regulation in *Burkholderia* have been unresolved.

When testing the stability of the W phenotype outside of biofilms, we noticed that W populations grown in planktonic cultures produced Smooth (S) colonies at a rate of approximately 1/500. These mutants, which were heritable suppressors, motivated this study of the genetic and evolutionary mechanisms that permit biofilm specialists to regain planktonic fitness. We predicted that the duration of time that W mutants had adapted to biofilm growth would influence their paths to S suppression. We used the classic design of evolving populations founded from multiple ancestors differing in their adaptive history (Travisano et al., 1995; Kryazhimskiy et al., 2014), and conducted a suppressor screen using experimental evolution and genome sequencing to determine what kinds of mutations, and in what genes, enable biofilm specialists to subsequently adapt in a planktonic environment. We were specifically interested in whether this phenotypic suppression involved mutations that quantitatively modulated prior biofilm adaptations or rather occurred by qualitative LOF mutations that eliminated these adaptations. Furthermore, we investigated whether the ecological consequences of the W→S switch in multiple environments varied with evolutionary history, and predicted that mutants derived from more adapted ancestors would exhibit greater tradeoffs associated with suppression.

Here we present evidence suggesting that the length of biofilm adaptation limits the number of pathways by which SCV’s may evolve to restore niche breadth and balance biofilm and planktonic growth. More derived W ancestors tended to produce S mutants by secondary *wsp* mutations that reduced biofilm production but retained high fitness in each of the experimental environments. In contrast to our prediction, these mutants produced a discrete phenotypic switch with limited pleiotropic consequences. On the other hand, S mutants from more naïve ancestors revealed more systematic tradeoffs that largely restored the ancestral phenotype and eliminated any prior fitness gains in the biofilm environment. Furthermore, the mutations producing the S phenotype also altered biofilm composition and the ability to disperse from the biofilm, revealing the likely target of selection. Finally, this work identifies a novel two-component pathway that we predict integrates Wsp-mediated signaling with the transcriptional activation of biofilm-related genes leading to the W phenotype. Using experimental evolution to conduct both a forward and reverse genetic screen therefore provided evidence of how *Burkholderia* may balance biofilm development as well as dispersal.

## MATERIALS AND METHODS

### ANCESTRAL STRAINS AND EXPERIMENTAL CONDITIONS

The *Burkholderia cepacia* complex (Bcc) is a group of genetically diverse pathogens capable of infecting both plants and humans; notably patients with CF or chronic granulomatous disease (CGD) are at elevated risk of infection from environmental or nosocomial exposure, and suffer increased morbidity and mortality (Mahenthiralingam et al., 2005; Vial et al., 2011). While the strain used for this study, *B. cenocepacia* HI2424, was obtained from an onion field rather than from a human infection, the strain type represents an epidemic clone (LiPuma et al., 2002). As described previously (Ellis and Cooper, 2010), this ancestor was marked with *lacZ* to enable competitively neutral, blue-white screening of plated mixtures during direct competitions.

**FIGURE 1:**
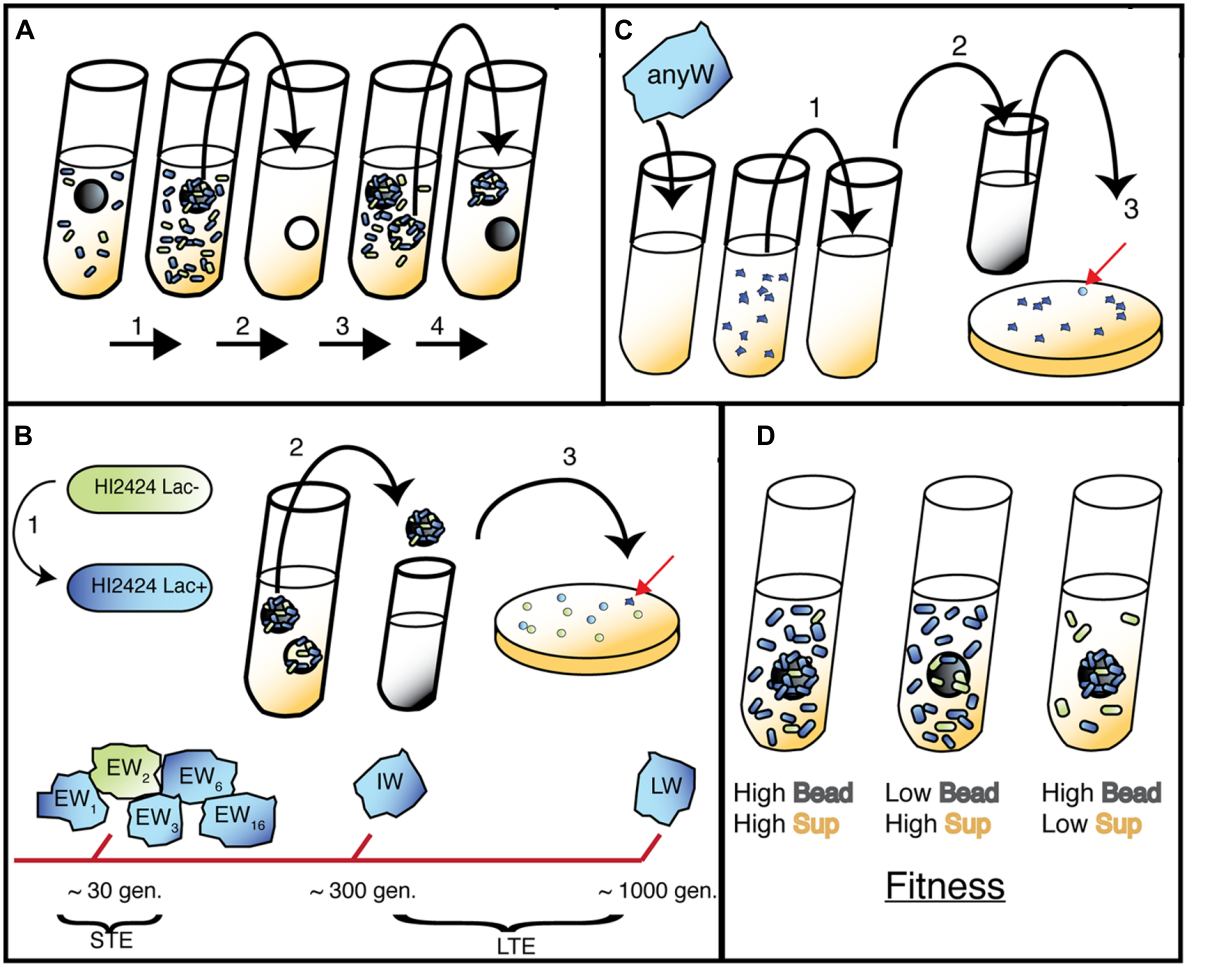
**Bead model of biofilm evolution.** **(A)** Bead-transfer method used in both evolution experiments. (1) A fresh bead is added to an inoculated tube containing marked (long-term evolution, LTE) or both marked and unmarked (short-term evolution, STE) WT populations and grown for 24 h. (2) Bead is colonized by biofilm cells and transferred to a fresh tube containing a sterile bead and growth media. (3) Bacteria disperse from original bead and attach to new bead. (4) New bead is selected and process repeats; note that 48-h bead is archived to sample representative population. **(B)** (1) Lac+ strain used in LTE and STE experiments, Lac- used in STE only. (2) Following daily transfer of colonized bead, remaining 48 h bead was removed and vortexed in PBS. (3) Populations were plated and screened for different morphologies related to adaptive traits (red arrow indicates a novel W mutant). Early Wrinkly (EW) mutants were detected at ∼30 generations, while Intermediate Wrinkly (IW) and Late Wrinkly (LW) were detected at ∼300 and 1000 generations. **(C)** Isolation of Smooth mutants. 1. W colonies were serially passaged in planktonic environment for three days. (2) Samples were diluted and plated. (3) Smooth colonies were detected and isolated. **(D)** Predicted fitness effects of adaptive mutants (blue cells) in sup and bead environment, with three outcomes presented: from left to right, early colonization and rapid dispersal; rapid dispersal but delayed colonization; early colonization and slow dispersal.

Although this *lac*Z-marked WT represents the original ancestor to all mutants described herein, our mutants were generated via a pair of independent evolution experiments conducted previously in our laboratory. As illustrated in **Figure 1**, the mutants produced in our suppression study were isolated from both short-term evolution (STE; Cooper et al., 2014) and long-term evolution (LTE) experiments (Traverse et al., 2013). The general approach, summarized below, was followed for both evolution experiments and has been described previously (Poltak and Cooper, 2011): ancestral clones were revived from frozen stock and grown for 24 h on a roller drum (RD; New Brunswick TC-7 model) at 37°C in 5 mL tryptic soy (T-soy; Fisher) broth using 18 mm × 150 mm test tubes. 50 μL of vortexed broth was subcultured in 5 mL M9 minimal media supplemented with 3% galactose (3% GMM) containing a sterile 7 mm polystyrene bead (American Education Products) and incubated at 37°C on the RD for 24 h. For the LTE experiment, a single Lac+ clone was inoculated into growth media. The bead was transferred daily to a new tube with fresh media and an oppositely marked bead, thus selecting for populations that were capable of daily biofilm formation and dispersal. Subsequent STE experiments were altered such that oppositely marked ancestral populations were mixed to identify beneficial mutants through marker divergence. Specifically, beads and biofilm colonists were transferred to a tube with phosphate buffered saline (PBS), vortexed, and marked (Lac+) and unmarked (Lac-) ancestors were added in equal 500 μL volumes to 4 mL of 3% GMM in tubes with a new bead, and incubated at 37°C for 24 h on the RD. Populations undergo approximately eight generations per day in this model. Populations were sampled by removing the 48-h bead, diluting 1:100,000 in PBS, and plating on half-strength T-soy agar plates containing X-gal. Plates were incubated for 24 h at 37°C, then at room temperature for 48 h. Unique colony morphologies were isolated and streaked to ensure selection of heritable traits. Freezer stocks were generated for each mutant isolate and stored in 8% DMSO at -80°C. Though a variety of phenotypes were generated from these STE and LTE experiments (Poltak and Cooper, 2011), only SCVs termed Wrinkly (W) were analyzed in this study.

### EXPERIMENTAL EVOLUTION AND MUTANT COLLECTION

Seven different W mutants were evolved to produce S variants, as follows. W cultures were streaked directly from freezer stock; a second streak was completed from a single isolate to ensure a homogenous clone was selected. This isolate was used to inoculate 5 mL of Tsoy broth, and incubated for 24 h at 37°C in the RD. 50 μL of the 24 h Tsoy broth was used to subculture a 5 mL tube containing 3% GMM without any bead, and incubated for 24 h. Transfers were repeated for 3 days in a planktonic environment, during which samples of broth were plated on strength Tsoy agar and examined for potential reversion of phenotype. S mutants were isolated from an agar plate, stocked at -80°C in 8% DMSO, and subjected to a bead-to-bead transfer in 3% GMM media for two days to ensure that the colony phenotype was heritable. Mutants listed with the same Wrinkly ancestor were generated from independent replicates; however, multiple mutants generated from a single culture were discarded from analysis due to an inability to verify their independent origin prior to sequencing.

### RELATIONSHIPS BETWEEN COLONY MORPHOLOGY AND MUCOIDY

All members of the Bcc have demonstrated capacity to produce a smooth, high EPS-producing phenotype termed “mucoid,” which is the result of enhanced expression of EPS (Zlosnik et al., 2008). The Mucoid phenotype is a result of enhanced expression of EPS. Following procedures described previously (Zlosnik et al., 2008), freezer stock of each Smooth was streaked on agar plates with minimal media consisting of 0.5 gL^-1^ yeast extract and 4 gL^-1^ mannitol to differentiate Mucoid (M) from Non-mucoid (N). Streaks were performed in duplicate, and observed at 24 and 48 h to confirm the production of EPS. Known positive and negative controls were obtained from a previous study that confirmed the presence and absence of EPS (Silva et al., 2011). Only isolates that produced copious amounts of EPS that diffused well outside of the streaked area were characterized as M.

### GENOME SEQUENCING

Genomes of all isolates were sequenced as described for both the STE (Cooper et al., 2014) and LTE (Traverse et al., 2013) experiments. Briefly, mutant isolates were grown on a bead, vortexed in PBS, and genomic DNA was extracted using the DNeasy Blood and Tissue Kit (Qiagen) protocol for Gram-negative bacteria. Libraries were prepared following standard protocols (Nugen). LTE genomes were sequenced by the Joint Genome Institute with an Illumina GAII using paired-end 36 bp reads (Traverse et al., 2013), while STE and all S mutant genomes were sequenced on the Illumina HiSeq2500 at the Hubbard Center for Genome Studies at the University of New Hampshire. Reads were mapped to the previously sequenced *B. cenocepacia* HI2424 reference genome (LiPuma et al., 2002) revealing > 100x coverage, for each clone. Mutations were identified using the breseq pipeline with default settings, focusing only on high-confidence mutations, although marginal calls or missing coverage were evaluated by visual inspection of aligned reads when present at high frequency or when the gene was mutated in more than one clone (Deatherage and Barrick, 2014).

### FITNESS ASSAYS

Methods for fitness assays were reported previously for both STE (Cooper et al., 2014) and LTE (Traverse et al., 2013) experiments, but now include a new environmental condition highlighted below. As previously, three- or fourfold replicate competitions were conducted between oppositely marked ancestor and mutants. All samples were revived from freezer stock in 5 mL Tsoy and incubated at 37°C for 24 h on a RD. 50 μL of Tsoy culture was added to 1 mL of PBS, vortexed, and 50 μL of the dilute sample was conditioned in 5 mL 3% GMM either with a bead or without (termed the planktonic environment) for 24 h on a RD at 37°C. For the bead environment, the single bead conditioned in GMM was removed with sterile forceps, transferred to 1 mL PBS and vortexed; the planktonic environment was completed by transferring 50 μL into the PBS dilution tube and vortexing. Competitions between evolved isolates and ancestral WT were conducted by adding 250 μL of mutant culture and 750 μL of WT to 4 mL GMM. Samples were vortexed, 100 μL of broth was removed and diluted 1:10,000 in PBS, and plated on Tsoy agar with X-gal. Cultures were incubated at 37°C in a RD for 24 h. Following incubation, planktonic tubes were vortexed and 50 μL of culture was added to 1 mL PBS and diluted; dilution samples were plated at both 1:10,000 and 1:100,000 on Tsoy agar with X-gal. Bead tubes followed a similar dilution protocol, with the bead and biofilm community being transferred to the PBS tube instead. A new environment, termed ‘sup’ for supernatant, was introduced in this experiment and represented the vortexed remains once the bead was removed from the *T* = 24 h tube. Similar to the planktonic culture, 50 μL of the broth remaining in the bead tube was transferred to a PBS tube, diluted, and plated. All plates were incubated for 24 h at 37°C and then 48 h at room temperature before colonies were enumerated. Colony forming units (CFUs) at *T* = 0 and *T* = 24 h were used to determine relative fitness as the selection rate constant, which is the difference (mutant – ancestor) in the natural logarithm of realized growth over 24 h (Lenski et al., 1991).

### BIOFILM AND EXOPOLYSACCHARIDE (EPS) ASSAYS

Biofilm production was measured via crystal violet absorbance (O’Toole, 2011). Briefly, freezer stock was revived in 5 mL of Tsoy, and grown overnight for 24 h at 37°C in a RD. Tsoy broth was added to 3% GMM to create a 5 mL solution with an optical density (O.D.) of 0.1, with 200 μL of diluted sample added to wells of a 96-well plate (Corning Costar). Four replicates were conducted for each treatment. Following 24 h of incubation at 37°C, biofilm production was measured by crystal violet staining, with 550 nm absorbance values recorded for all samples.

Relative EPS production was quantified following a hybrid of methods described previously (Ueda and Wood, 2009). This growth media is different than that used to measure biofilm production and quantify fitness, and was selected for its ability to differentiate EPS production among mutants. Isolate colonies were revived from freezer stock in 5 mL of Tsoy and grown overnight for 24 h at 37°C in a RD. Tsoy broth was added to respective 96-well plate containing 200 μL of mannitol media (MM) with 40 μg/mL Congo red dye such that all flasks had initial O.D. of 0.1. Inoculated wells were incubated for 24 h at 37°C, after which the contents of wells were pelleted in a centrifuge at 4,000 rpm for 10 min at 4°C. One hundred microliters of supernatant were transferred to a new 96-well plate, and contents were again centrifuged. Finally, 50 μL of this supernatant was added to a new 96-well plate, and absorbance was measured at 490 nm. Relative EPS production was reported as the difference in absorbance between blank (Congo Red and mannitol) and sample.

### STATISTICAL ANALYSES

Analysis of variance (ANOVA) and *post hoc* Tukey tests were performed with JMP software (JMP®;, Version 11.0. SAS Institute Inc., Cary, NC, USA, 1989–2007). A *P*-value of <0.05 was considered statistically significant. *Post hoc* comparisons between experimental factors were delineated by different letters if statistically different, and share the same letter if indistinguishable.

## RESULTS

### ISOLATION OF S MUTANTS FROM GENETICALLY DISTINCT W ANCESTORS

Mutants with a W phenotype, which exhibited nearly invariant high fitness in a model biofilm environment (Cooper et al., 2014), were significantly worse in a planktonic environment lacking a plastic bead for attachment (Figure S1). We predicted this tradeoff generated strong selection for mutants that restore planktonic fitness. Seven W mutants with different genotypes and evolutionary histories were each used to found several replicate populations that were passaged under planktonic conditions for 3 days and screened for colony variants. Of these W ancestors, five had previously evolved during biofilm selection for 32–64 generations and acquired single *wsp* mutations (Cooper et al., 2014), one evolved for 315 generations and acquired at least one secondary mutation in *rpoC,* and one W ancestor evolved for 1050 generations and acquired at least eight mutations (Traverse et al., 2013). Twenty mutants with S morphology from these different ancestors were collected and labeled ‘E’ for arising from an Early W ancestor, ‘I’ for arising from the Intermediate W ancestor, or ‘L’ for the Late ancestor. Once these phenotypes were confirmed to be heritable, the genomes of these mutants were sequenced to determine the genetic basis of suppression (**Table 1**) and to identify any additional mutations acquired during the 3 days of passaging in growth media (Table S1). Isolates were labeled according to their duration of evolution (Early-E, Intermediate-I, Late-L), colony morphology (W or S), production of EPS [Mucoid, M, or Non-mucoid (N)], and ancestor (numeral).

**Table 1:** Mutations predicted to produce Smooth (S) colonies from various Wrinkly (W) ancestors.

Ancestral wrinkly genotype	Predicted causative suppression mutation (smooth)
Name	Mutation	Locus	Gene name	Name	Mutation	Locus	Gene name	Mutant class
EW_1_	A452V	Bcen2424_3786	*wspA*	ESM_1.2_	D98N	Bcen2424_1436	RR/ transcript. activator	I
				ESM_1.4_	Δ2 bp, 330-331, frameshift	Bcen2424_1436	RR/ transcript. activator	I
EW_2_	D652N	Bcen2424_3791	*wspE*	ESM_2.1_	Q444*	Bcen2424_3786	*wspA*	II
				ESM_2.5_	R202H	Bcen2424_1436	RR/ transcript. activator	I
EW_3_	S285W	Bcen2424_3786	*wspA*	ESM_3.3_	Δ53 bp, 355-407	Bcen2424_3790	*wspD*	II
				ESM_3.4_	Δ1,714 bp (-105/-122)	Bcen2424_1436	RR/ transcript. activator	I
				ESM_3.6_	L205R	Bcen2424_1436	RR/ transcript. activator	I
EW_6_	S726L	Bcen2424_3791	*wspE*	ESM_6.1_	Δ1 bp, 716	Bcen2424_4210	beta mannosidase	III
				ESM_6.2_	Δ8 bp, 573-580	Bcen2424_3788	*wspC*	II
EW_16_	V389A	Bcen2424_3786	*wspA*	ESM_16.1_	Insertion, 463-702	Bcen2424_3790	*wspD*	II
				ESM_16.4_	Insertion, 838-1134	Bcen2424_3785	*wspHRR*	II
IW	I196N	Bcen2424_3786	*wspA*	ISM_1_	E195*	Bcen2424_3785	*wspHRR*	II
				ISN_3_	Δ5 bp, 1696-1700	Bcen2424_4198	hypothetical protein	III
				ISM_4_	S291*	Bcen2424_3786	*wspA*	II
				ISM_8_	A506P	Bcen2424_3786	*wspA*	II
				ISM_9_	K55N	Bcen2424_3791	*wspE*	II
LW	A407V	Bcen2424_3786	*wspA*	LSM_2_	Δ2 bp, 834-835	Bcen2424_3786	*wspA*	II
				LSN_3_	G275R	Bcen2424_4204	glycosyl transferase	III
				LSM_4_	I197T	Bcen2424_3785	*wspHRR*	II
				LSM_5_	S191L	Bcen2424_1436	RR/ transcript. activator	I


Multiple S mutants were collected during experimental evolution of eight different W ancestors of varying genotype (*wsp* mutation) and evolutionary history (generations of biofilm selection, E , Early; I , Intermediate, L, Late). EPS production described in name (M, Mucoid EPS producer; N, Non-mucoid, EPS deficient). Predicted wrinkly suppressor mutations fell into three classes: loss-of-function (LOF) mutations in a response regulator/transcriptional activator (Class I), secondary LOF *wsp* mutations (Class II), or mutations affecting polysaccharide production (Class III). Mutations annotated with an asterisk (*) involve premature stop codons.

### MUTATIONS SUPPRESSING THE W PHENOTYPE FORM THREE CLASSES

All seven W ancestors used in this study possess a mutation in either the *wspA* or *wspE* genes (**Table 1**; Traverse et al., 2013; Cooper et al., 2014). Of the 20 suppression mutants of these W ancestors, 11 isolates acquired a secondary *wsp* mutation that likely converted the colony phenotype from wrinkly to smooth, but only three of these new mutations occurred in the same *wsp* gene as the initial *wsp* mutation. The remaining nine S mutants involved non-*wsp* mutations. Given the strong parallelism among independent mutants, we confidently attribute missense mutations and a single deletion in an undescribed two-component transcriptional regulator, *Bcen2424_1436* (termed “1436” moving forward), to be the genetic cause of six suppression events. Notably, all five SNP’s within 1436 clustered within putative REC and OmpR domains (Figure S2). Though *wsp* and *1436* together accounted for 17 of the 20 suppression mutations, three additional mutations in gene clusters associated with polysaccharide production illustrated additional paths by which the W phenotype can revert to the ancestral smooth colony type. These three mutations did not occur in the same operon, however, nor do their annotations suggest that they contribute exclusively with EPS production (Winsor et al., 2008). We collectively refer to these three genes as *PPA* for “Polysaccharide Production-Associated,” given their putative functions.

Importantly, the types of W suppression mutations (*wsp*, *1436*, or *PPA*) were not equally distributed among isolates of different selective histories. Among the I and L mutants that evolved from a more derived ancestor (i.e., following a greater period of biofilm selection), six of nine acquired a secondary suppression mutation in *wsp* and only one mutant featured a mutation in 1436. Suppressor mutations identified in the ES isolates that evolved from W ancestors with more limited histories of biofilm selection occurred as many times in *1436* as in *wsp*, demonstrating alternative evolutionary paths to S. It should be noted that the frequency with which any of these S suppression phenotypes occurred was rare – often several plates with over 200 colonies of W phenotypes would have just a single smooth-colony mutant among them. Though the rate of mutation to the S phenotype was not quantified, E, I, and L ancestors each produced S mutants with low frequency (data not shown).

Although all S mutants acquired only a single mutation in either *wsp*, 1436, or PPA, additional missense or non sense mutations were identified in six of 21 isolates (Table S1). Three isolates had single SNPs, two isolates acquired a pair of unique SNPs each, and one isolate contained a SNP and a 2-base-pair missense substitution. The mutated genes were diverse, including glycosyltransferases, restriction enzymes, transcriptional regulators, and flagellar assembly proteins. These additional mutations may be beneficial and could contribute to the fitness of S mutants. More likely, however, is that these additional mutations may be effectively neutral and the presence of these mutations can be attributed to genetic hitchhiking with the three classes of suppression mutations, especially given that most S mutants contained only a single causative mutation.

### FITNESS EFFECTS OF SUPPRESSOR MUTANTS DIFFER BY GENOTYPE AND AMONG ENVIRONMENTS

Mutations in the same gene or operon may nonetheless produce different phenotypes or growth affinities, especially if secondary mutations are not neutral, so we quantified fitness in three environments by direct competitions between S mutants and the original HI2424 ancestor. These environments were the selective biofilm environment with a plastic bead in a test tube, a planktonic environment lacking a bead, and (new to this study) a third condition termed ‘sup’ to indicate the supernatant phase of the bead-containing environment. The sup condition provides a unique opportunity to quantify the tendency of each mutant to disperse from an established biofilm or grow in the immediate suspension. Because the bead condition exclusively measures attached cells, and the sup condition the cells in the planktonic phase suspended above the biofilm, comparing fitness in the sup and bead conditions enables predictions about the life history strategy of an isolate. For example, mutants with high fitness in both the biofilm and sup environments suggest a strategy of early attachment and early dispersal. In contrast, a mutant with high fitness in the biofilm but low fitness in the sup condition suggests later dispersal timing, or alternatively, reduced ability to disperse. Lastly, mutants with low fitness in the biofilm but high fitness in the sup condition could be late or low biofilm formers and/or early dispersers. These assays of fitness across environments coupled with defined genotypes provided the opportunity to explore genetic mechanisms involved with distinct biofilm life history strategies (**Figure 1**).

**FIGURE 2:**
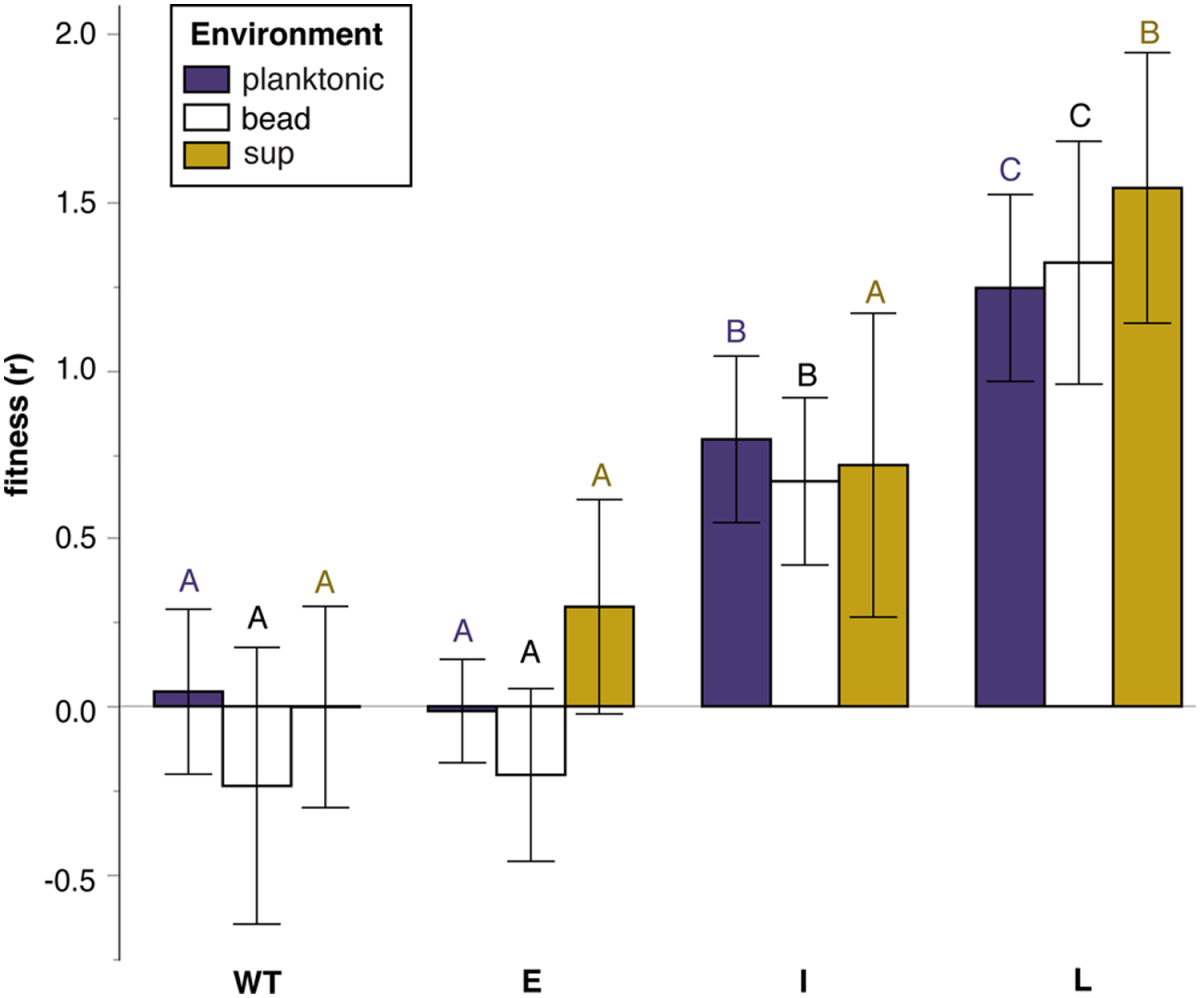
**Relative fitness (selection rate, *r* ) of S mutants grouped by the duration of selection of theirW ancestor in a biofilm environment: Early (E) ~30 generations, Intermediate (I) ~300 generations, Late (L) ~1000 generations**. Error bars are 95% CI. Tukey post hoc tests were used for pairwise comparisons. Unique letters within environments reflect significant differences.

**FIGURE 3:**
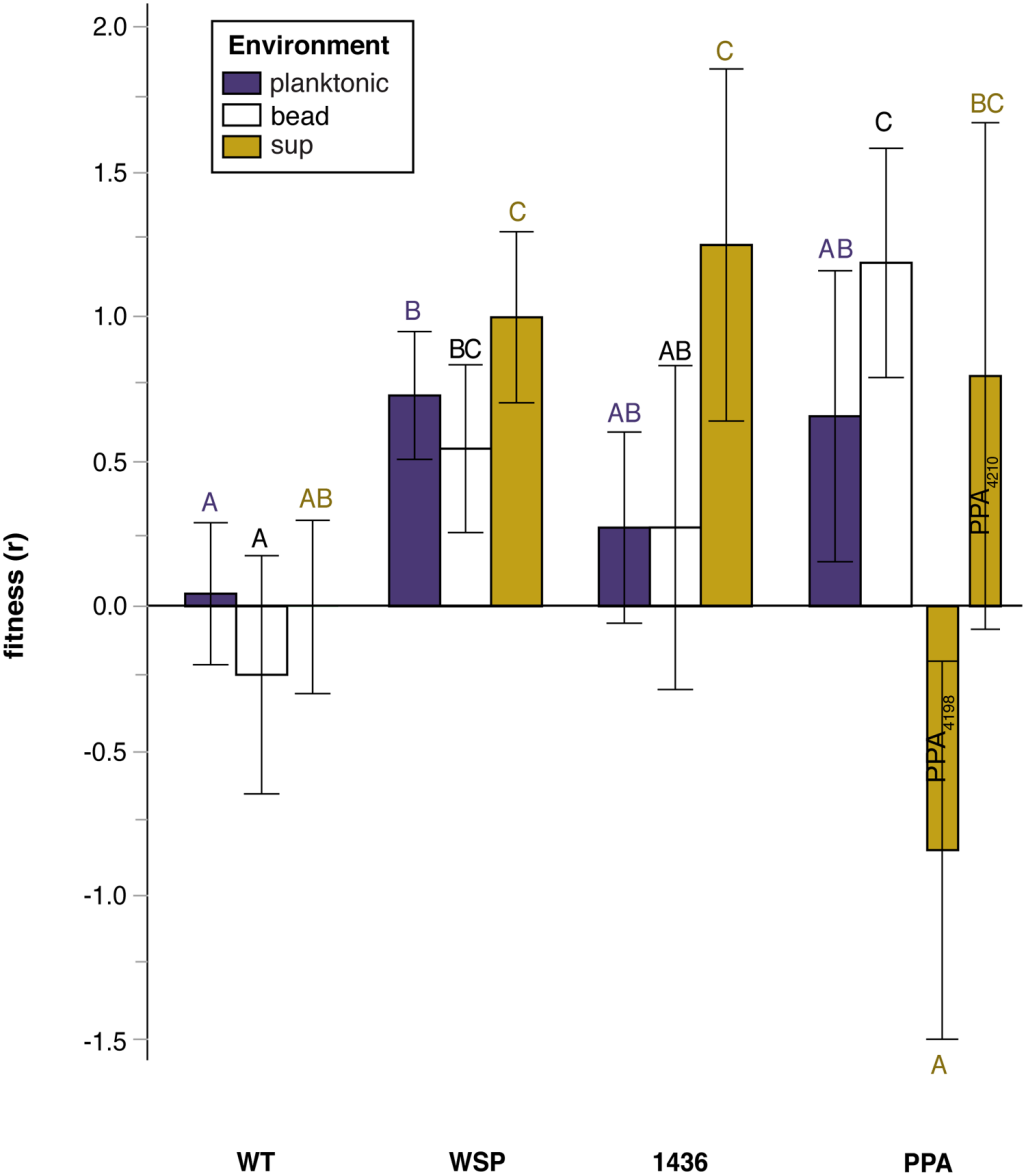
**Relative fitness (selection rate, *r*) in three environments of mutants grouped by their suppression mutation class: wild-type (WT), two-component transcriptional regulator (1436), wrinkly-spreader operon (*wsp*), and putative polysaccharide production associated (PPA) genes.** Note that mutation class PPA has been split for the ‘sup’ environment to distinguish between populations by duration of prior biofilm selection: ISN_3_ (intermediate) compared to LSN_3_ (late). Tukey *post hoc* tests were used for pairwise comparisons, with unique letters reflecting significant differences. Error bars are 95% CI of the mean.

Fitness effects of S mutants varied significantly with the duration of adaptation of the W parental genotypes in each of the three environments tested (**Figure 2**; Tables S2 and S3). The fitness of ES mutants was similar to WT in all environments, suggesting that the suppression mechanism largely

restored WT fitness from the high biofilm-specific benefit of the W phenotype (Cooper et al., 2014). However, IS and LS mutants displayed significant fitness increases (**Figure 2**). Specifically, LS mutants were significantly adaptive in all three environments, whereas IS mutants were adaptive in both bead and planktonic environments. These results revealed that the relative fitness of S suppressor mutants varied both as a function of duration of biofilm adaptation and the environment in which they compete. Although mutants derived from ancestors that had previously evolved in the biofilm model for longer periods tended to be better adapted in all environments, the two non-mucoid isolates (ISN_3_ and LSN_3_) were outliers in the sup environment (**Figure 3**). These findings suggested that mutational mechanism as well as selective history could explain fitness variance.

We thus evaluated whether mutational class rather than the duration of biofilm selection explained fitness differences among suppressors (**Figure 3**; Table S3). Relative fitness varied significantly among mutational classes, with each group more fit than WT in at least one environment; however, only suppressors with a secondary mutation in *wsp* were significantly more fit across all environments, including the planktonic phase. The sup environment revealed the greatest variation in fitness among mutation classes: *wsp* and *1436* mutants were more fit than WT, but *PPA* mutants that were non-mucoid were less fit than WT.

### BIOFILM PHENOTYPES ASSOCIATED WITH SUPPRESSION

**FIGURE 4:**
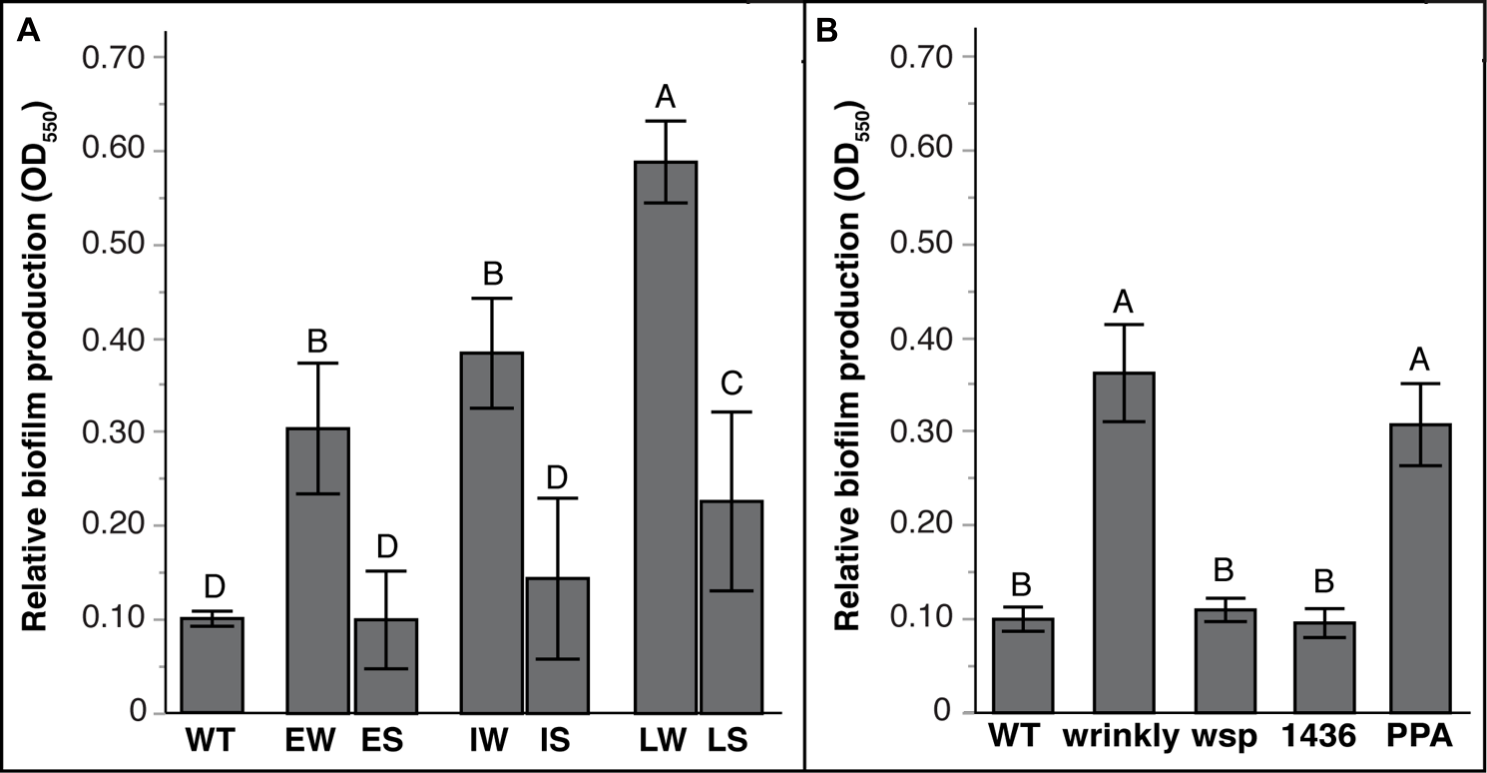
**Biofilm production varies with duration of biofilm adaptation and by mutation class.** Mean biofilm production with 95% CI shown. Tukey *post hoc* tests were used for pairwise comparisons, with unique letters reflecting significant differences. **(A)** Mean biofilm production by selective history of ancestors: Early Wrinkly (EW), Early Smooth (ES), Intermediate Wrinkly (IW), Intermediate Smooth (IS), Late Wrinkly (LW), Late Smooth (LS). **(B)** Biofilm production by mutation class: wild-type (WT), two-component transcriptional regulator (1436), *wsp* operon, and putative PPA genes.

As predicted by the tradeoff model between biofilm and planktonic growth, mean biofilm production varied significantly between W and S mutants (**Figure 4**; Tables S4 and S5). Prior studies of mutants from long-term selection in our model demonstrated that biofilm production tended to increase with further generations of selection (Poltak and Cooper, 2011; Cooper et al., 2014). Here, biofilm production of S suppressor mutants also varied with prior selective history (**Figure 4A**): LS mutants produced more biofilm than WT and other mutants, but IS and ES mutants produced similar biofilm as WT (Table S4). More evidently, biofilm output differed among mutational classes: both *wsp* and *1436* suppression mutants produced much less biofilm, whereas *PPA* mutations maintained biofilm productivity equal to wrinkly ancestors (**Figure 4B**; Table S5). This suggests that *PPA* mutations are not critical for biofilm production, whereas the two-component transcriptional regulator *1436,* like *wsp,* is directly involved in the activation of biofilm production.

Another clinically important phenotype that appeared to change with the shift from W to S colony types was their mucoidy (Silva et al., 2011; Zlosnik et al., 2011). When grown on agar plates containing mannitol, Mucoid (M) clones will produce significant quantities of exopolysaccharide (EPS) whereas Non-mucoids (N) will appear opaque and produce little or no visible liquid on the plate. Within 48 h, WT HI2424 and each W ancestor were capable of producing robust EPS. While 18 of 20 suppression mutants also formed copious quantities of EPS, two suppression mutants, ISN_3_ and LSN_3_, were non-mucoid. These two isolates each acquired unique suppression mutations in genes associated with EPS production (Fazli et al., 2013). Interestingly, a frameshift mutation in the other affected *PPA* gene, *Bcen2424_4210*, was sufficient to suppress the W phenotype without the loss of EPS production. This alternative compensatory mutational path was identified in mutants derived from EW, IW, and LW ancestors. These results illustrate that EPS production may be retained independently of the W phenotype, but mutations in at least two genes were sufficient to reduce both EPS production and suppress the W phenotype.

**FIGURE 5:**
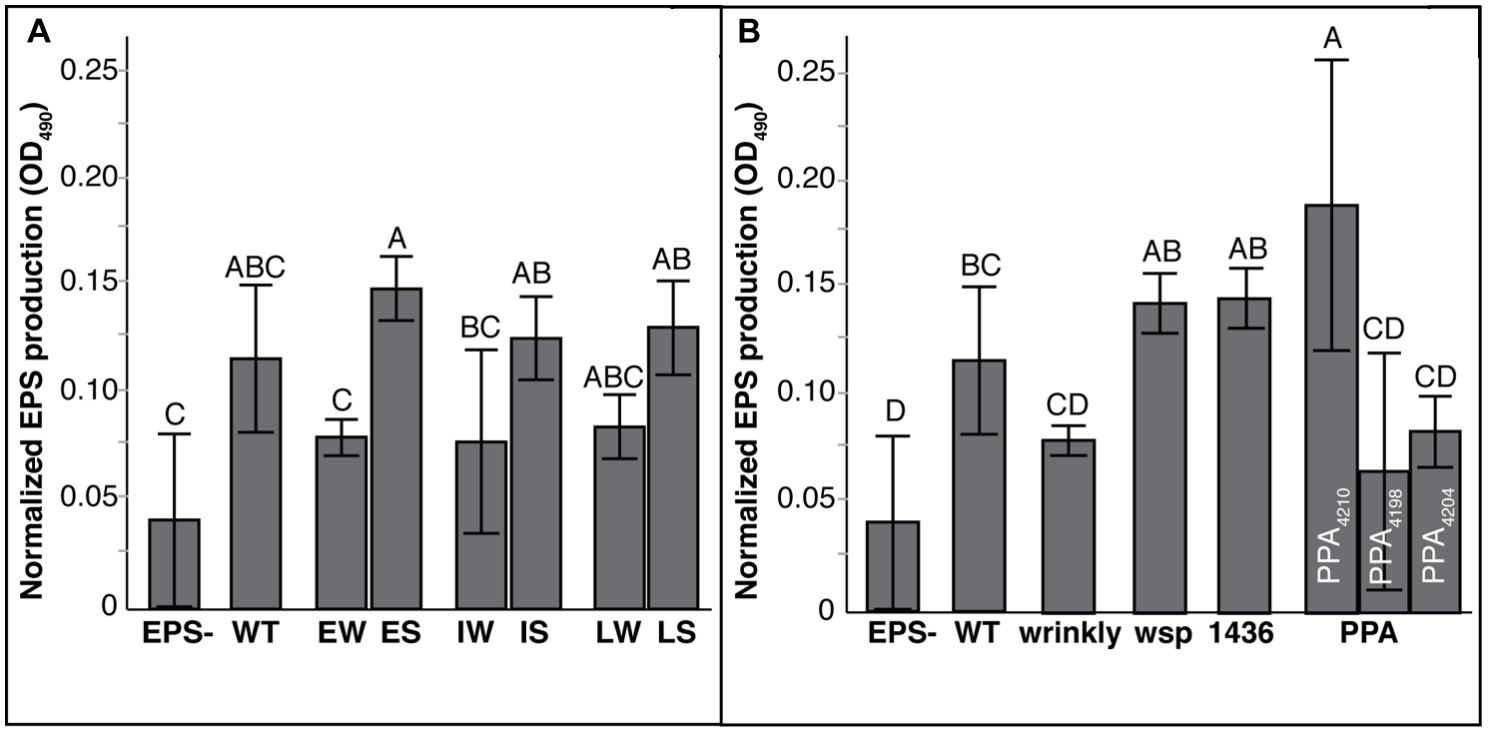
**Exopolysaccharide (EPS) production differs by mutation class but not prior history of biofilm adaptation.** EPS production is expressed as the mean difference between the blank and supernatant of culture, with 95% CI. Tukey *post hoc* tests were used for pairwise comparisons, with unique labels reflecting significant differences. **(A)** EPS production by generation of biofilms: Early Wrinkly (EW), Early Smooth (ES), Intermediate Wrinkly (IW, Intermediate Smooth (IS), Late Wrinkly (LW), Late Smooth (LS). **(B)** EPS production by mutation class: wild-type (WT), wrinkly, two-component transcriptional regulator (1436), *wsp*, and putative PPA genes. PPA mutants are plotted separated to show significant differences between mutations affecting putative EPS loci (4198 and 4204) and non-EPS loci (4210).

The extent of EPS production produced by these mutants also correlated with the mutational mode of suppression (**Figure 5**; Tables S6 and S7). *PPA* mutants were distinct as a group and among mutants within this group: mutants ISN_3_ and LSN_3_ both acquired mutations in a newly described EPS cluster (Fazli et al., 2013) and produced EPS at significantly lower levels than *1436* and *wsp* mutants (**Figure 5B**). *PPA* mutant ESM_6.2_ acquired a frameshift mutation in a gene putatively encoding a beta-mannosidase that is outside that putative EPS cluster, and produced the greatest amount of EPS among all mutants. To our surprise, and for uncertain reasons, W mutants produced less EPS than S mutants with *1436* or *wsp* mutations (**Figure 5**).

## DISCUSSION

It is now widely accepted that the biofilm lifestyle is the dominant mode of growth and survival for most bacteria (O’Toole et al., 2000; Karatan and Watnick, 2009). For decades, however, research has been primarily directed toward study of microbes in well-mixed planktonic cultures. Therefore, the understanding of bacterial physiology in naturalistic conditions may be tenuous at best. Fortunately, thee last 15 years have seen many genetic screens yield substantial insight into processes governing biofilm community assembly (Kuramitsu et al., 2007; Ryder et al., 2007; Irie and Parsek, 2008; McDougald et al., 2012; Fazli et al., 2014) and integrity (Spiers and Rainey, 2005; Borlee et al., 2010). Such models have demonstrated complexity in biofilm regulation, suggesting that these opportunistic pathogens exhibit multiple overlapping signaling networks that influence the fine-tuning of the composition and expression of biofilm components (Petrova and Sauer, 2009; Fazli et al., 2014).

It seems probable, though, that planktonic and biofilm growth as they are typically modeled in the laboratory represent extreme conditions. Rather, bacterial populations may fluctuate between motile and sessile states at a range of frequencies. Understanding of how bacteria genetically adapt to growth in a biofilm, but then successfully disperse to grow planktonically or to re-initiate biofilm formation, is much more limited, largely because of a lack of methods to realistically probe the phenotypes under selection in these variable environments. Most experiments have involved genetic screens for losses of biofilm production or planktonic growth. These studies tend to find mutations that block transitions between extreme phenotypes, but these findings lack nuance and tend not to find gain-of-function mutations. However, when transposon mutagenesis and the less biased method of experimental evolution were combined to probe *P. fluorescens* biofilm production, mutations in relatively few genes explained much of the variation in biofilm assembly (McDonald et al., 2009; Callahan et al., 2014). These studies suggest that perhaps only a handful of gene products are under positive selection for the production of certain biofilms. Yet little research has investigated how bacteria might subsequently adapt to a more motile lifestyle, as would be required to disperse and colonize a new environment. Moreover, suppression studies of mutants produced by transposons cannot realistically capture these lifestyle reversals because the initial step inherently prevents back mutations. Therefore, the experimental evolution approach presented here provides greater potential for investigating the genes and pathways that regulate biofilm growth as well as dispersal to resume planktonic growth.

### ADAPTIVE GENETIC MECHANISMS THAT RESTORE DISPERSAL CAPACITY FOR BIOFILM SPECIALISTS

We previously characterized the genetic and ecological origins of the Wrinkly (W) phenotype in *B. cenocepacia* from many independently evolved mutants with varying histories of adaptation to biofilm growth on plastic beads (Traverse et al., 2013; Cooper et al., 2014). The cause of the W phenotype was nearly always a single missense mutation in either the *wspA* or *wspE* gene. Despite this evidence, the downstream targets of the *wsp* cluster and the causes of the wrinkly phenotype in *B. cenocepacia* have been unsolved, which motivated this study for mutations that would suppress this phenotype. Here we show that three classes of mutations can suppress the W phenotype and restore planktonic fitness and the ancestral colony phenotype. The first group, secondary mutations in the *wsp* operon, was most expected, although the locations of these suppressor mutations suggest that residues predicted to be involved in WspA dimerization (ISM_8_), WspE phosphorelay (ISM_9_), or WspHRR kinase activity (LSM_4_) are essential for Wsp system function (**Table 1**). Next, we discovered mutations in a novel transcriptional regulator, *Bcen2424_1436*. Importantly, all five SNP mutations occurred within predicted functional activation and response domains, demonstrating that losses of function in residues predicted to mediate phosphorylation or DNA binding produce similar phenotypes (**Table 1**; Figure S2). In addition, mutations in three other genes implicated in polysaccharide synthesis produced the S phenotype from W ancestors, indicating that the W phenotype likely depends more on downstream effects of Wsp signaling rather than the Wsp proteins directly. Importantly, nearly all of these suppressor mutations, which include nine indels and three premature stop codons, likely produce qualitative losses of function (**Table 1**). Evidently, the strong experimental selection to restore planktonic fitness favored LOF mutations that would be expected to limit subsequent evolution of W-type biofilm specialists.

The striking molecular parallelism among *1436* mutants illustrates the power of experimental evolution to identify functional roles for predicted proteins of unknown function. Among the six S 1436 mutants, one substitution occurred five amino acids from a KPV motif known to be a conserved part of the phosphate receiver domain, while four others each contained mutations at conserved residues associated with DNA binding (Marchler-Bauer et al., 2013; Figure S2). No other mutations occurred in these genotypes, which offers a clear prediction that the S phenotype results from either blocking the stimulation of *1436* by phosphorylation, or by eliminating its function as a DNA-binding transcriptional activator. This *1436* gene is found in many *Burkholderia* species and is always accompanied by a putative membrane-associated two-component response regulator with a histidine kinase domain, named Bcen2424_1438 in strain HI2424. This study therefore enables the additional prediction that a previously undefined two-component system, in which Bcen2424_1438 receives a periplasmic signal that it transduces to *1436* to activate transcription, plays a central role in modulating biofilm attachment and dispersal in *Burkholderia*.

**FIGURE 6:**
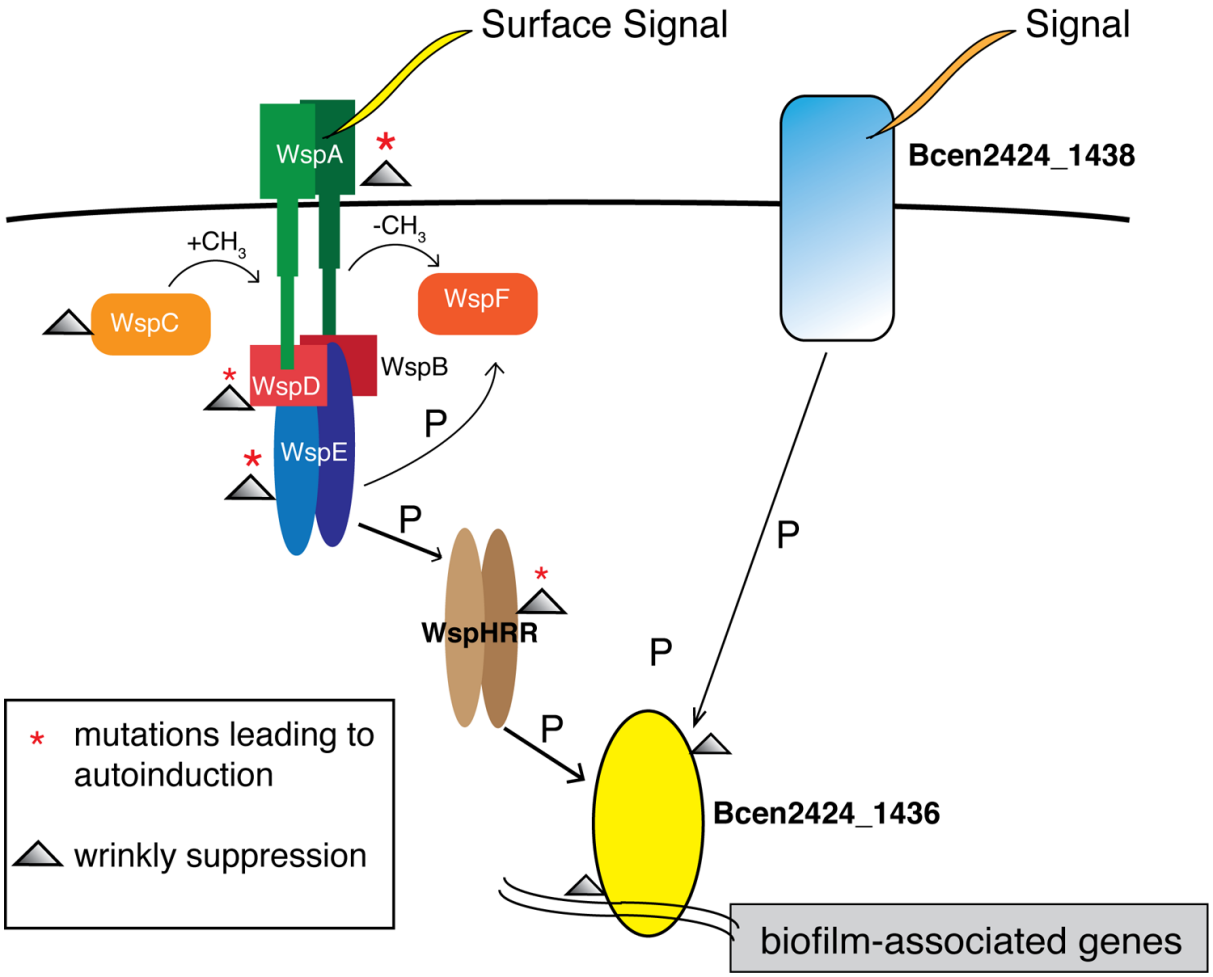
**Model of biofilm regulation in *Burkholderia cenocepacia* building upon the Wsp model in *Pseudomonas*.** Surface signaling at WspA leads to a phosphorylation cascade, activating WspHRR. W mutants in this study possessed mutations in *wspA* and *wspE*, leading to autoinduction of this process; S mutants possessed secondary mutations that also clustered in *wsp* or in *Bcen2424_1436*, as highlighted by symbols in the legend. We predict that the DNA-binding response-regulator Bcen2424_1436 is phosphorylated by WspHRR, which stimulates biofilm production by transcriptional activation. As part of a predicted two-component system, the Bcen2424_1436 protein also likely receive signals from a transmembrane sensor histidine kinase, Bcen2424_1438, which has conserved domains matching a periplasmic domain, a PAS fold, and a HATPase domain.

The possible interactions between this *1438/1436* two-component system and the *wsp* system are less clear, but this study provides considerable circumstantial evidence. Again, recalling that all W ancestors began with a single *wsp* mutation, 17 of 20 S suppressors were caused by secondary mutations in either *wsp* or *1436.* Importantly, S mutants with either *wsp* or *1436* mutations produced similar phenotypes and fitness profiles across all environments (**Figure 3**), which indicates that that these gene products likely cooperate in a single path regulating biofilm development in our experimental model. We therefore hypothesize that *1436,* which has a N-terminal CheY-like response regulator and a C-terminal winged helix-turn-helix DNA binding effector domain, is directly responsible for activating transcription of biofilm associated processes, and is activated via phosphorylation of the *wsp* pathway by *wspHRR* (**Figure 6**). We previously predicted, based on very strong similarity to the *Pseudomonas* *wsp* system (Cooper et al., 2014), that a phosphorylation cascade is initiated upon surface growth being detected via WspA, leading ultimately to *wspHRR* activation. Here we predict that the phosphate transfer from *wspHRR* to *1436* completes the cascade and allows 1436 to bind and activate the transcription of genes that initiate biofilm production. However, the fact that *1436* is part of an operon containing a periplasmic protein, *Bcen2424_1438*, with its own predicted Chase2 and PAS sensory domains, suggests that alternative phosphorelay paths may activate overlapping biofilm-related genes. This 1438/1436 two-component system is broadly similar to EnvZ/OmpR and CreB/C in multiple well-studied bacterial genomes (Winsor et al., 2008), but is not the reciprocal best match of either of these sensor kinases that mediate central metabolism and play roles in both resistance and biofilm formation (Prigent-Combaret et al., 2001; Zamorano et al., 2014). Thus, the Wsp system may serve as the master regulator of surface detection in *B. cenocepacia* HI2424 as it does in *Pseudomonas* (O’Connor et al., 2012), but *1436* may integrate both Wsp signaling and additional sensed inputs. Clearly, further work detailing the transcripts activated by *1436* as well as the kinases that activate it will clarify the complex regulatory networks that govern *Burkholderia* biofilm development.

This study also illustrates parallels and differences between the Wsp system in *Burkholderia* and in *Pseudomonas*, in which the proximate cause of the W morphology is cellulose overproduction (Spiers and Rainey, 2005), and the ultimate source involves mutations that de-repress the diguanylate cyclase (DGC) WspR, leading to elevated levels of cyclic-di-GMP (Hickman et al., 2005; McDonald et al., 2009). In addition to Wsp, numerous other systems have been found in a wide range of bacteria to regulate biofilm formation via c-di-GMP levels (McDougald et al., 2012; Srivastava and Waters, 2012; Fazli et al., 2014). In contrast, the *Burkholderia* Wsp system does not contain a cognate DGC, so if altered cyclic di-GMP production is also the ultimate selective mechanism, the regulatory connection is indirect. This sensory system may therefore have been coopted in *Burkholderia* to activate transcription more directly by phosphorelay, with the DGC module being replaced by a histidine kinase that we called *wspHRR* (Cooper et al., 2014; **Figure 6**). Given our prediction that ancestral W mutants had constitutively active Wsp systems, a logical means of suppression would involve secondary *wsp* mutations, which we found 11 times. Six other mutations affected 1436, which demonstrate that its function is essential for Wsp signaling. However, the three mutations affecting genes related to the proximate cause of the smooth morphology, polysaccharide synthesis, tell a similar story to that of *Pseudomonas*. In both of these biofilm-forming bacteria, there appear to be relatively few regulatory ways to produce a hyper-biofilm producing specialist, but more ways involving both regulation and structural components to undo this specialization.

### EFFECTS OF HISTORY ON ADAPTATION TO A NEW ENVIRONMENT: PATHS AND TRADEOFFS

Another goal of this study was to evaluate the contingent effects of evolutionary history on adaptation, and to test the prediction that greater biofilm specialization would constrain adaptation to planktonic conditions. The results of this study generally support the notion that an organism’s life history is important but not sufficient to predict its evolutionary trajectory. We found that the suppression of an evolved W phenotype and its associated low fitness in planktonic conditions can arise by mutations in three major gene classes and furthermore, that the history of biofilm adaptation may limit the potential number of targets available to revert these phenotypes. While increased specialization appears to limit adaptive paths in a different environment, mutants that manage such a step appear nonetheless to benefit from their accumulated mutational history. Indeed, only S mutants evolved from more derived W ancestors were fitter than WT across all environments, in effect broadening their niche in fluctuating biofilm and planktonic environments and showing little evidence of tradeoffs. Overall, mutants ofW clones isolated following LTE in our model were fittest in nearly all comparisons (**Figure 2**), suggesting that their past selection in fluctuating biofilm environment favored genotypes that were broadly beneficial.

This evidence that past adaptive gains in the long-term biofilm model were robust to mutations affecting the W phenotype led us to test for fitness epistasis between *wsp*-associated mutations and other mutations found in the I and L genotypes (Khan et al., 2011; Flynn et al., 2013). Although the experimental design was not originally designed to evaluate this possibility and is therefore not ideal, we found no evidence of interactions for fitness among genotypes either having or lacking the set of mutations acquired during LTE (Table S8). This finding suggests the remarkable possibility that biofilm-adapted genotypes may gain or lose the SCV phenotype of W with little or no effect on other adaptations. If SCV phenotypes may generally evolve without influencing other adaptations, this could explain their frequent recovery from diverse biofilm-associated infections (Haussler et al., 2003; Starkey et al., 2009; Lee et al., 2011; Silva et al., 2011; Mayer-Hamblett et al., 2014).

Despite consistent benefits of prior adaptations in our laboratory system, S genotypes nevertheless varied in phenotypes and fitness depending on their mutational path adapting to a planktonic lifestyle. For instance, mutants of *1436* were most fit in the supernatant environment, reflecting early dispersal. We predict that these *1436* mutants fail to activate transcription of various biofilm-related genes and effectively fail to respond to the Wsp signal of surface attachment, and thus disperse too early to be sampled on the bead. In contrast, fitness effects of *wsp* double-mutants are consistently elevated relative to WT across all three environments and follow a similar logic: whereas the initial *wsp* mutation leads to constitutive attachment and high biofilm fitness, we predict the second suppressor mutation blunts this surface-attachment signal and restores the ability to disperse and achieve high fitness in the sup environment. This conclusion is complicated by the fact that more *wsp* double-mutants arose in more derived genotypes that were originally more fit. Despite these distinctions, the similar fitness profiles among *1436* and *wsp* mutants (**Figure 3**), as well as their similar reductions in biofilm and EPS production (**Figure 4 and 5**), support our model that they are integrated in a pathway initiating biofilm production (**Figure 6**). Thus, interrupting the signal of being attached to a surface may be a major step facilitating the transition from a biofilm-adapted to planktonic-adapted mode.

In general, the S suppression mutants produced consistently lower levels of biofilm and EPS regardless of prior adaptive history (**Figure 4 and 5**). These biofilm traits contrast with fitness in attaching to the bead, in which more evolved mutants may nevertheless benefit from other adaptive mutations in spite of reduced biofilm and EPS production. However, PPA mutants produced distinct fitness and phenotype effects that suggest the proximate cause of wrinkly colonies. Two PPA mutants enhanced fitness in attaching to the bead but reduced fitness in the supernatant of the biofilm. These mutants affected homologs of a newly described EPS cluster, *Bcam1330-1341* (Fazli et al., 2013), reduced EPS production relative to WT, and became non-mucoid. The nature of these mutations is illuminating: one involved a deletion in a hypothetical protein that may be responsible for cleaving the final product for secretion into the matrix, while the other involved a missense mutation in a putative glycosyl transferase (Fazli et al., 2013). The third *PPA* mutation, a frameshift in beta-mannosidase, actually increased EPS production and this mutant was more fit in the supernatant environment; we predict this mutation disrupts mannose degradation and enhances polysaccharide synthesis. The fact that these mutations converted the wrinkly colony phenotype into smooth types suggests strongly that that the affected loci are downstream targets of the *wsp/1436* regulon and major constituents of the biofilm. They may also play roles in balancing biofilm life history, as EPS synthesis may be required for dispersal in addition to biofilm assembly. Given the purported hygroscopic properties of these polysaccharides, an absence or even partial reduction in EPS production may substantially increase the viscosity of the matrix and prevent dispersal (McDougald et al., 2012). Further, because the *PPA* mutants retain the ancestral, constitutively activated Wsp system, the inability of the N types to form EPS could be combining with greater synthesis of other polymers, such as cellulose, that may prevent dispersal.

To conclude, this study began as a survey of the mutations that explained the relatively frequent evolution of smooth mutant colonies from wrinkly, SCV ancestors during growth in planktonic culture. We wondered whether the causes of these smooth suppressor mutants differed with the ancestral genotype and the duration of prior biofilm selection on these wrinkly ancestors; furthermore, we predicted that greater biofilm selection would generate greater tradeoffs across environments for these suppressors owing to stronger antagonistic pleiotropy. We paired short-term experimental evolution with high-throughput sequencing to survey the mechanisms of planktonic adaptation and suppression of the W phenotype, and conducted a range of assays of fitness in multiple environments and of biofilm-associated phenotypes. The evolved mutations identified mechanisms by which multiple previously uncharacterized genes contribute to the balance of biofilm formation and planktonic growth. Similarly, the fitness assays contribute to a narrative that shows how these various genotypes and phenotypes may evolve in response to a common selective force. Evolutionary history was generally predictive of fitness and biofilm production, but not for EPS synthesis. The specific mutational paths taken appear to be more predictive still, such that despite any advantageous mutational heritage derived from prolonged biofilm adaptation, a single base pair substitution in a few targets appears sufficient to dramatically alter phenotype, polysaccharide production, and fitness across a range of environments.

These findings may have broader implications for the evolutionary genetics of microbial populations. Perhaps most striking, the suppression of more adapted W mutants disproportionately involved secondary *wsp* mutants, which implies the evolution of constraint on alternative genetic pathways. Nevertheless, this constraint did not produce greater tradeoffs; rather, it appears that selection in the new planktonic environment limited the sampling of beneficial mutations to those producing few if any costs across environments. Perhaps with continued adaptation in one environment, the distribution of effects of beneficial mutants found in a new environment becomes increasingly skewed toward direct suppressors and revertants that undo prior specializing mutations. Consequently, genotypes relatively naïve in the first environment may be compensated by a range of mutations in the second, but not so in the case of a more derived genotype. Therefore, with increasing history of selection in a single environment, options for the evolutionary return trip may become few indeed.

## Conflict of Interest Statement

The authors declare that the research was conducted in the absence of any commercial or financial relationships that could be construed as a potential conflict of interest.
