# Supplementary material for: There and back again: consequences of biofilm specialization under selection for dispersal
Source: Front Genet. 2015 Feb 11;6:18. doi: 10.3389/fgene.2015.00018 (PMC4324302; doi:10.3389/fgene.2015.00018)
Supplement: Supplementary file 1 [file Data_Sheet_1.PDF]

Figure S1. Relative fitness during planktonic phase growth of experimentally evolved W mutants with various Wsp genotypes, as described in the legend. Just as the biofilm fitness of these mutants is uniformly positive in a model of competitive attachment to a plastic bead (Cooper et al., 2014), planktonic fitness is uniformly negative.

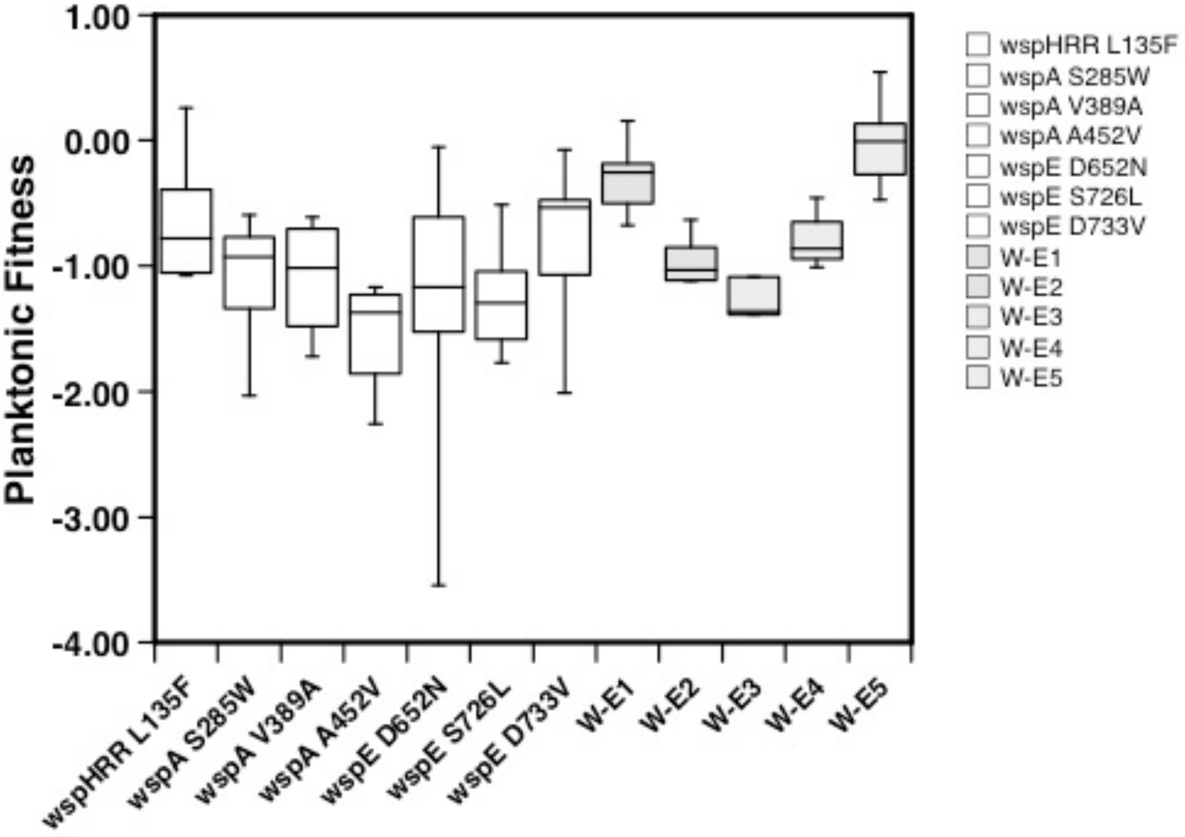

Figure S2. Schematic of mutations affecting the Bcen2424_1436/1437/1438 locus, a putative two-component system that when mutated suppresses the wrinkly colony phenotype.

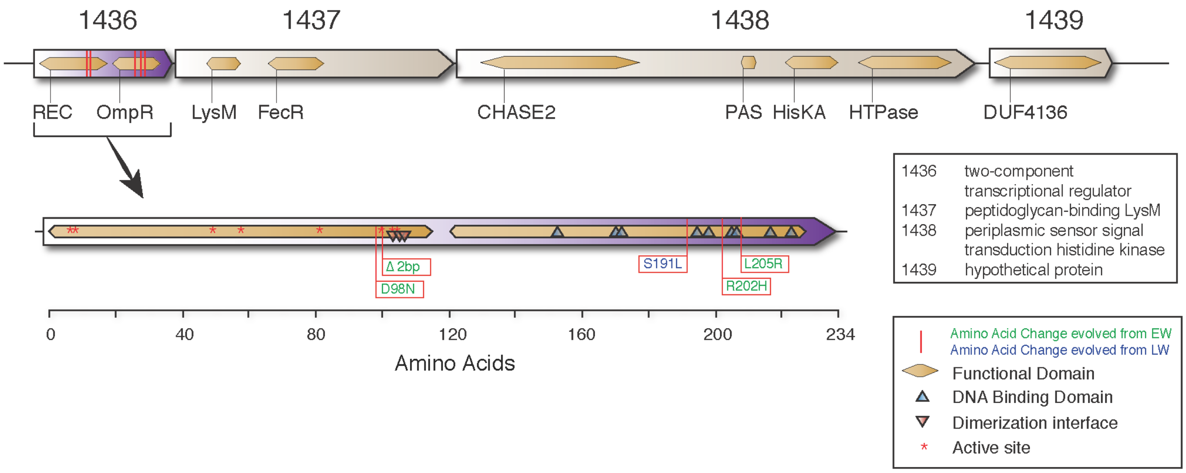

Table S1. Additional Smooth mutants. High-confidence mutations reported for locations not thought to be related to SCV phenotype reversion. S mutants not listed did not exhibit any additional mutations other than those reported in Table 1. Additional ancestral wrinkly mutations occurred in both Iw and Lw (Traverse et al., 2013); no high-confidence mutations were detected in any Ew isolates. Notable protein associations were determined by Strings v9.1 (Francheshini et al., 2013); genes names as reported from *Burkholderia* database (Winsor et al., 2008).

**Suppression Mutant Genotype**				
**Name**	**Mutation**	**Locus**	**Gene name**	**Putative Associations**
**ESM1.4**	A178T	Bcen2424_0027	type III restriction enzyme	
**ESM2.5**	V178G	Bcen2424_3441	hypothetical protein	In operon with histidine kinase, two-component regulator, and MltA-interacting protein MipA;
**ESM3.3**	A359P	Bcen2424_1170	glycosyl transferase family protein	Associated with ydjC family protein and radical SAM domain containing protein
	A146P	Bcen2424_4105	hypothetical protein	Operon containing NmrA family protein, dTDP-4-dehydrorhamnose reductase, and several other glycosyltransferases
**ISM4**	A137P	Bcen2424_0020	alkylhydroperoxidase	adjacent to extracellular sigma factor (ECF) SigJ
	A140K	Bcen2424_4376	sigma-54 dependent transcriptional regulator	Mutation in the Walker-B motif of the AAA+ ATPase domain
**LSM4**	D246E	Bcen2424_4039	hypothetical protein	
**LSM5**	V170V	*fliH*	flagellar assembly protein H	Motility-associated
	D104D	bcen2424_4295	acyl-CoA synthetase	

Table S2. Relative fitness between groups across environments. ANOVA and pairwise comparisons of the treatment variables i) duration of biofilm adaptation (E = early, I = intermediate, L = late) and ii) suppression mutation class (*wsp*, 1436, or PPA). Note that for a pair of analyses in the sup condition, PPA was split into the two respective loci due to significant variation between the pair.

Environment	ANOVA		Competition			
			Competing pair	Relative Fitness (r)		
	F	p		Δ r	SE	p <
planktonic	[3,84] = 30.18	< 0.0001	L vs. WT	1.20	0.19	0.0001
			L vs. E	1.25	0.15	0.0001
			L vs. I	0.45	0.15	0.05
			I vs. WT	0.75	0.19	0.001
			I vs. E	0.80	0.14	0.0001
bead	[3,71] = 23.90	< 0.0001	L vs. WT	1.55	0.25	0.0001
			L vs. E	1.54	0.21	0.0001
			L vs. I	0.65	0.19	0.01
			I vs. WT	0.90	0.24	0.001
			I vs. E	0.89	0.20	0.01
sup	[3,67] = 10.42	< 0.0001	L vs. WT	1.54	0.31	0.0001
			L vs. E	1.24	0.28	0.001
			L vs. I	0.82	0.26	0.05

planktonic	[3,84] = 4.13	< 0.01	*wsp* vs. WT	0.68	0.23	0.05
bead	[3,71] = 5.96	< 0.01	*PPA* vs. WT	1.42	0.35	0.001
			PPA vs. *1436*	0.91	0.34	0.05
			wsp vs. WT	0.78	0.29	0.05
sup	[4,67] = 9.34	< 0.0001	*wsp* vs. WT	1.00	0.27	0.01
			wsp vs. 1436	1.84	0.38	0.0001
			1436 vs. WT	1.25	0.35	0.01
			1436 vs. PPA_{4198}	2.09	0.43	0.0001
			PPA_{4204} vs. PPA_{4198}	1.64	0.48	0.01

Table S3. Pairwise differences in mean relative fitness between mutant isolate and wild-type (WT). Mutants are named by age (I = intermediate, L = late), appearance (S = smooth), and production of EPS on mannitol plate (M = mucoid, N = Non-mucoid). Fitness expressed as the selection rate (r), with standard error (SE) and probability values from t-tests presented.

Mutant vs. WT	Relative Fitness (r)		
	mean	SE	p
ISN_3	- 0.851	0.28	< 0.001
LSM_2	1.41	0.28	< 0.05
LSN_3	0.95	0.28	0.24
LSM_4	2.17	0.32	< 0.001
LSM_5	1.98	0.32	< 0.01
LSN_3	1.67	0.36	< 0.01
LSM_4	1.43	0.40	< 0.05
LSM_5	1.57	0.31	< 0.05

Table S4. Pairwise differences in biofilm production between mutants grouped by evolutionary history in the biofilm, as described in the text. Pairwise differences were evaluated by t-tests, and variance among all groups was assessed by ANOVA: $F_{[107,7]} = 42.59$, $p < 0.0001$

Pair		Relative Biofilm production		
		Δ mean	SE	P <
LW	ES	0.486	0.036	0.0001
LW	WT	0.486	0.049	0.0001
LW	IS	0.443	0.038	0.0001
LW	EPS-	0.387	0.048	0.0001
LW	LS	0.361	0.038	0.0001
LW	EW	0.283	0.038	0.0001
LW	IW	0.203	0.048	0.005
IW	ES	0.284	0.036	0.0001
IW	WT	0.283	0.048	0.0001
IW	IS	0.240	0.038	0.0001
IW	EPS-	0.184	0.048	0.01
IW	LS	0.158	0.038	0.005
EW	ES	0.203	0.020	0.0001
EW	WT	0.203	0.048	0.0001
EW	IS	0.159	0.023	0.0001
EW	LS	0077	0.024	0.05
LS	ES	0.126	0.020	0.0001
LS	WT	0.125	0.038	0.05
LS	IS	0.082	0.023	0.05

Table S5. Pairwise differences in biofilm production between mutant classes, as described in the text. Pairwise differences were evaluated by t-tests, and variance among all groups was assessed by ANOVA: $F_{[107,5]} = 56.89$, $p < 0.0001$

Pair		Relative Biofilm production		
		Δ mean	SE	P <
W	*1436*	0.267	0.021	0.0001
W	WT	0.263	0.038	0.0001
W	*wsp*	0.253	0.018	0.0001
W	EPS-	0.164	0.037	0.0005
PPA	*1436*	0.212	0.025	0.0001
PPA	WT	0.208	0.040	0.0001
PPA	*Wsp*	0.198	0.023	0.0001

Table S6. Pairwise differences in EPS production between mutants grouped by mutation class, as described in the text. Pairwise differences were evaluated by t-tests, and variance among all groups was assessed by ANOVA:a $F_{[122,7]} = 15.30$, $p < 0.0001$

Pair		Relative EPS production		
		Δ mean	SE	P <
PPA_{4210}	EPS-	0.149	0.027	0.0001
PPA_{4210}	PPA_{4198}	0.126	0.027	0.0005
PPA_{4210}	W	0.110	0.020	0.0001
PPA_{4210}	PPA_{4204}-	0.107	0.027	0.0005
PPA_{4210}	WT	0.074	0.024	0.05
1436	EPS-	0.105	0.020	0.0001
1436	PPA_{4198}	0.082	0.020	0.005
1436	W	0.067	0.010	0.0001
1436	PPA_{4204}	0.063	0.020	0.05
wsp	EPS-	0.103	0.020	0.0001
wsp	PPA_{4198}	0.079	0.020	0.005
wsp	W	0.064	0.009	0.0001
wsp	PPA_{4204}	0.061	0.020	0.05
WT	EPS-	0.076	0.024	0.05

Table S7. Pairwise differences in EPS production between mutants grouped by evolutionary history in the biofilm, as described in the text. Pairwise differences were evaluated by t-tests, and variance among all groups was assessed by ANOVA: $F_{[122,7]} = 9.28$, $p < 0.0001$.

Pair		Relative EPS production		
		Δ mean	SE	P <
ES	EPS-	0.108	0.022	0.0001
ES	IW	0.076	0.022	0.05
ES	EW	0.070	0.011	0.0001
IS	EPS-	0.085	0.023	0.01
IS	EW	0.047	0.013	0.05
LS	EPS-	0.090	0.023	0.005
LS	EW	0.051	0.014	0.01

Table S8. Post-hoc pairwise tests following ANOVA of fitness effects of mutation classes under A) planktonic growth B) biofilm conditions or C) the sup environment. Classes are defined by the initial causative mutation (wsp), subsequent biofilm-adaptive mutations (other), and then the mutation producing suppression. Letters denote statistically similar groupings. Mutants featuring "other" adaptive mutations are generally grouped, regardless of the mutational cause of suppression, suggesting that fitness effects of these mutations do not interact with the suppression mutation.

A

**Genotype**			**Mean fitness**
wsp + other + 1436	A		1.568681
wsp + other + wsp	A	B	1.057921
wsp + other + PPA	B	C	0.655929
ancestor	C	D	0.043664
wsp + wsp	D		0.015939
wsp + 1436	D		-0.016657

B.

**Genotype**			**Mean fitness**
wsp + other + PPA	A		1.185399
wsp + other + wsp	A		0.900857
wsp + other + 1436	A	B	0.741196
wsp + 1436		B	0.003720
ancestor		B	-0.236296
wsp + wsp		B	-0.363336

C.

**Genotype**			**Mean fitness**
wsp + other + 1436	A		1.983937
wsp + other + wsp	A		1.373603
wsp + 1436	A	B	0.755656
wsp + other + PPA		B	0.051002
wsp + wsp		B	0.019777
ancestor		B	-0.001871
